# Rapid Blood Culture Diagnostics for Ambulatory Management of Uncomplicated Gram-Negative Bloodstream Infections

**DOI:** 10.1093/ofid/ofaf075

**Published:** 2025-02-12

**Authors:** Martina Boda, Kristina E Rokas, Christina F Yen, Daniel J Diekema, Nicholas J Mercuro

**Affiliations:** Department of Pharmacy, MaineHealth Maine Medical Center, Portland, Maine, USA; Department of Pharmacy, MaineHealth Maine Medical Center, Portland, Maine, USA; Division of Infectious Diseases, MaineHealth Maine Medical Center, Portland, Maine, USA; Division of Infectious Diseases, MaineHealth Maine Medical Center, Portland, Maine, USA; Department of Pharmacy, MaineHealth Maine Medical Center, Portland, Maine, USA

To the  Editor—Traditional automated susceptibility testing methods, which take on average 3 days to finalize, can prolong the hospitalizations of patients who are clinically stable with gram-negative bacilli (GNB) bloodstream infections (BSIs) [1, 2]. A rapid blood culture identification and phenotype technology, Accelerate Pheno, performs organism identification via automated fluorescence in situ hybridization and phenotypic antimicrobial susceptibility testing via morphokinetic cellular analysis for a number of organisms included on a predetermined panel. Ventres et al implemented Accelerate Pheno to provide organism identification and susceptibilities within 8 hours of blood culture positivity, facilitating antibiotic optimization, switch to an oral agent, and hospital discharge in GNB BSI [1, 3, 4]. Patients with GNB BSI are a heterogeneous population; many have fewer comorbidities, experience rapid source control, and reach clinical stability sooner than others [1, 2, 5, 6]. Recent observational data suggest that early discharge and ambulatory management of some BSIs are safe [6]. There is an opportunity to leverage rapid phenotype reports when treating patients who are stable and have early clinical responses to therapy.

At Maine Medical Center, positive blood culture results are reviewed daily by an infectious diseases pharmacist; general therapeutic advice is provided about antimicrobial selection, route, and duration, with management and disposition decisions determined by the clinician. This single-center retrospective cohort (2021–2023) assessed adults with uncomplicated GNB BSI who were treated as outpatients or admitted <48 hours with antibiotic therapy guided by rapid identification and phenotype. During the study period, all blood cultures with GNB on initial Gram stain were tested with Accelerate Pheno. Measured outcomes were treatment failure (defined as requiring antibiotic treatment for isolation of the same organism at the prior source within 30 days), all-cause mortality at 90 days, and 30-day readmissions. Patients who did not have documented failure, readmission, or mortality documented in the electronic health record were presumed to have a successful outcome.

We screened 262 adults with GNB BSI and length of stay (LOS) <5 days. Exclusion criteria were met in 182 patients: LOS >48 hours, transfer from outside hospital, polymicrobial BSI and/or inability for Accelerate Pheno to identify organism, or inpatient mortality/discharge to hospice. Among the 80 patients who were included, admitted patients (n = 59) were often discharged from their primary emergency department visit (32.8%) and called back to be admitted for BSI management; among those not admitted (n = 21), 14 (66.7%) returned to the emergency department within a median 7 hours (IQR, 3–13) for reevaluation. The time from positive culture result to rapid phenotype result was a median 8 hours (IQR, 7.5–9); cultures were finalized by a median 96 hours (IQR, 46–115). The most common source of infection was urinary (90%), followed by intra-abdominal (6.3%). All patients received at least 1 intravenous (IV) antibiotic dose, most frequently ceftriaxone (65%) or cefepime (12.8%); the first antibiotic was active in 91% of cases. The median LOS was 1.6 days (IQR, 1.1–1.8). After discharge, 10 patients (12.5%) required a change in oral therapy based on rapid diagnostic results. There were 3 categorical discrepancies identified between the rapid phenotypic and final blood culture result; however, this was not clinically impactful as none of the patients were prescribed an antimicrobial for which the discrepancy was present. In 2 cases, the rapid phenotype indicated *Escherichia coli* with a ciprofloxacin minimum inhibitory concentration ≤0.25 mg/L, with finalized minimum inhibitory concentrations of 1 to 2 mg/L; another case was *Enterobacter cloacae* with rapid susceptibilities reporting resistance to cefepime and piperacillin-tazobactam but susceptible in the final automated susceptibility report. No patients died within 90 days; the frequency of readmissions was low (Table 1). Seven patients (8.7%) met treatment failure criteria, with 6 for suspected urinary tract infection recurrence. One patient with a recent prostatectomy experienced a recurrent *Klebsiella pneumoniae* BSI despite a 10-day course of ciprofloxacin; no nidus of infection was identified on repeat imaging.

**Table 1.** Population Characteristics and Outcomes

	Median (IQR) or No. (%)		
	Total (n = 80)	Discharged From ED Without Admission (n = 21)	Admitted for <48 h (n = 59)
Demographics			
Age, y	67 (51–77)	67 (44–76.5)	67 (54–78)
Sex: male	37 (46.3)	11 (52.4)	26 (44.1)
Baseline characteristics			
Charlson Comorbidity Index	3 (1–5)	3 (0–4)	3 (2–5)
Pitt bacteremia score	0 (0–1)	0 (0–0)	0 (0–1)
SBP <90 mm Hg	1 (1.3)	0 (0)	1 (1.7)
Tmax >38 °C	33 (41.3)	5 (23.8)	28 (47.5)
WBC, 10^3^ cells/mL			
Initial	13.2 (10.4–18.1)	11.5 (8.9–17.1)	13.4 (10.9–18.3)
Discharge	10.7 (6.3–14.7)	11.2 (7.0–16.0)	10 (6.1–14.3)
Causative pathogen			
*Escherichia coli*	61 (76.3)	18 (85.7)	43 (72.9)
*Klebsiella* spp	8 (10)	1 (4.8)	7 (11.9)
*Enterobacter* spp	7 (8.8)	0 (0)	7 (11.9)
Source of bacteremia			
Urinary	72 (90)	19 (90.4)	53 (89.8)
Intra-abdominal	6 (6.3)	1 (4.8)	5 (6.8)
Other	3 (1.7)	1 (4.8)	2 (3.4)
Source control procedure			
Needed^a^	17 (21.3)	2 (9.5)	15 (25.9)
Achieved	7 (41.2)	2 (100)	5 (33.3)
Total antibiotic duration, d	9 (8–12)	9 (8–11)	9 (8–13)
Oral antibiotic			
β-lactam	28 (35)	12 (57.1)	16 (27.1)
TMP-SMX	26 (32.5)	6 (28.6)	20 (33.9)
Fluoroquinolone	26 (32.5)	3 (14.3)	23 (39)
30-d outcome			
Treatment failure	7(8.7)	2 (9.5)	5 (8.5)
All-cause readmission	7 (8.8)	1 (4.8)	6 (10.2)
Infection-related ED or hospital readmission	6 (7.5)	1 (4.5)	5 (8.5)

Abbreviations: ED, emergency department; SBP, systolic blood pressure; Tmax, maximum temperature; TMP-SMX, trimethoprim-sulfamethoxazole; WBC, white blood count.

^a^Procedures were related to 14 cases of stenting for ureteral/renal stones, 1 endoscopic retrograde cholangiopancreatography, 1 seroma postprostatectomy, and 1 dental extraction.

Infectious diseases clinicians and antimicrobial stewards are frequently asked, “How long should a patient undergo IV therapy prior to oral switch?” However, neither prospective nor randomized trials suggest that any specific duration of IV lead-in is required. In a trial of adults with pyelonephritis and complicated urinary tract infection (about 40% with positive blood culture results) who were undergoing oral ciprofloxacin (no IV lead-in) vs IV ciprofloxacin, there was no difference in outcomes [7]. Two randomized trials comparing IV with oral administration in GNB BSI required at least a 72-hour IV lead-in before oral switch; both achieved shortened LOS and similar clinical outcomes [8, 9]. Switching to orals should be based on clinical response, a known infection source, the ability to take/absorb an oral antibiotic, and the availability of an organism and site-appropriate agent.

In our study, adults with GNB BSI who were discharged within 48 hours or treated as outpatients rarely experienced complications, comparable to findings by Casado et al, who also observed low revisits and mortality in patients who were ambulatory with BSI; in other literature, 30-day readmissions are in the 10%–20% range [1–6]. This is likely due to the selection bias in patients who are stable and deemed to be safely treated as ambulatory by the clinician, as demonstrated by Charlson Comorbidity Index and Pitt bacteremia score. The availability of rapid susceptibilies to oral agents gives further confidence in making this decision. However, treatment outcomes were evaluated through review of patient charts, which limits the ability to identify treatment failure, readmissions, or other follow-up data if patients sought care outside the health system. A power analysis was not pursued, as the effect size of the intervention could not be assessed without a comparator arm; thus, we cannot confirm whether admission requirements or hospital LOS was affected by rapid diagnostic results. With a limited sample size and low event rate, additional statistical analyses to determine associations with failures and readmissions were not performed.

With these limitations in mind, our findings may support that a subgroup of adults who are stable with GNB BSI from a known source (primarily urinary) and have an early clinical response to empiric therapy could safely switch to an oral antibiotic based on rapid phenotype to facilitate early discharge or prevent admission. These data support the conclusions from Ventres et al that rapid phenotypic results can optimize patient outcomes when coupled with antimicrobial stewardship [1]. These hypotheses warrant confirmation in a prospective randomized trial.

## Notes

***Financial support*.** The research funding granted to D. J. D. was not utilized in this study. No funding by external bodies was received to complete this work.
